# The microtubule quartet protein SNAP1 in *Trypanosoma brucei* facilitates flagellum and cell division plane positioning by promoting basal body segregation

**DOI:** 10.1016/j.jbc.2023.105340

**Published:** 2023-10-12

**Authors:** Thiago Souza Onofre, Kieu T.M. Pham, Qing Zhou, Ziyin Li

**Affiliations:** Department of Microbiology and Molecular Genetics, McGovern Medical School, University of Texas Health Science Center at Houston, Houston, Texas, USA

**Keywords:** *Trypanosoma brucei*, microtubule quartet, hook complex, flagellum inheritance, cytokinesis

## Abstract

The unicellular protozoan *Trypanosoma brucei* has a single flagellum that is involved in cell motility, cell morphogenesis, and cell division. Inheritance of the newly assembled flagellum during the cell cycle requires its correct positioning, which depends on the faithful duplication or segregation of multiple flagellum-associated cytoskeletal structures, including the basal body, the flagellum attachment zone, and the hook complex. Along the flagellum attachment zone sites a set of four microtubules termed the microtubule quartet (MtQ), whose molecular function remains enigmatic. We recently reported that the MtQ-localized protein NHL1 interacts with the microtubule-binding protein TbSpef1 and regulates flagellum inheritance by promoting basal body rotation and segregation. Here, we identified a TbSpef1- and NHL1-associated protein named SNAP1, which co-localizes with NHL1 and TbSpef1 at the proximal portion of the MtQ, depends on TbSpef1 for localization and is required for NHL1 localization to the MtQ. Knockdown of SNAP1 impairs the rotation and segregation of the basal body, the elongation of the flagellum attachment zone filament, and the positioning of the newly assembled flagellum, thereby causing mis-placement of the cell division plane, a halt in cleavage furrow ingression, and an inhibition of cytokinesis completion. Together, these findings uncover a coordinating role of SNAP1 with TbSpef1 and NHL1 in facilitating flagellum positioning and cell division plane placement for the completion of cytokinesis.

*Trypanosoma brucei*, a protozoan parasite and the causative agent of sleeping sickness in humans and nagana in cattle in sub-Saharan Africa, has a motile flagellum which additionally determines cell morphology, defines the cell division plane, and likely mediates intercellular communications (1, 2, 3). The flagellum, composed of a microtubule-based axoneme and an extra-axonemal structural termed the paraflagellar rod, is nucleated from the basal body, a centriole-like structure composed of a mature basal body (mBB) and a pro-basal body (pBB) (4, 5, 6), whose duplication represents the first cytoskeletal event of the trypanosome cell cycle. The flagellum exits the cell body through the flagellar pocket, and is attached to the cell membrane *via* a specialized cytoskeletal structure termed the flagellum attachment zone (FAZ) (7). During the early stages of the cell cycle in *T. brucei*, a new flagellum is assembled from the newly matured pBB, and two new pBB are assembled next to the two mature basal bodies (6). Subsequently, the new mBB–pBB pair and the new flagellum make a rotational move by re-locating from the anterior side of the old mBB–pBB pair and the old flagellum toward their posterior side (8). Further, the elongation of the new flagellum during the following cell cycle stages coordinates with the migration of the new mBB–pBB pair toward the posterior portion of the cell, leading to the segregation of the duplicated mBB–pBB pairs and other flagellum-associated cytoskeletal structures (9). Upon successful cell division, each daughter cell inherits a single copy of the flagellum and its associated cytoskeletal structures (9).

The flagellum-associated cytoskeletal structures include the hook complex, which is originally termed the bilobed structure (10), the flagellar pocket collar (FPC) (11), the microtubule quartet (MtQ), and the FAZ (12, 13, 14, 15, 16, 17, 18, 19) (Fig. 1*A*). The hook complex is a hairpin-like structure consisting of a fishhook-like structure marked by TbMORN1 and a bar-shaped structure termed the centrin arm, which is marked by two centrin proteins, TbCentrin4 and TbCentrin2, and sits along the shank part of the fishhook-like structure (20, 21). The hook part of the fishhook-like structure sits at the top of the FPC, which is composed of the ring-forming protein TbBILBO1 (22), wraps around the flagellum, and runs alongside the MtQ (Fig. 1*A*). Embedded between the shank part of the fishhook-like structure and the centrin arm is the proximal end of the intracellular FAZ filament (20), which further extends toward the anterior tip of the cell to maintain the attachment of the flagellum (12, 14, 15). Alongside the intracellular FAZ filament runs the MtQ (23), which is originated between the mBB and the pBB, traverses the FPC, and extends toward the anterior cell tip (11) (Fig. 1*A*). Inhibition of the duplication or segregation of these flagellum-associated cytoskeletal structures impairs flagellum positioning, disrupts flagellum attachment, and inhibits cell division (12, 13, 14, 15, 22, 24, 25). However, the control mechanisms for the duplication and the segregation of these cytoskeletal structures remain understudied and poorly understood.

**Figure 1:**
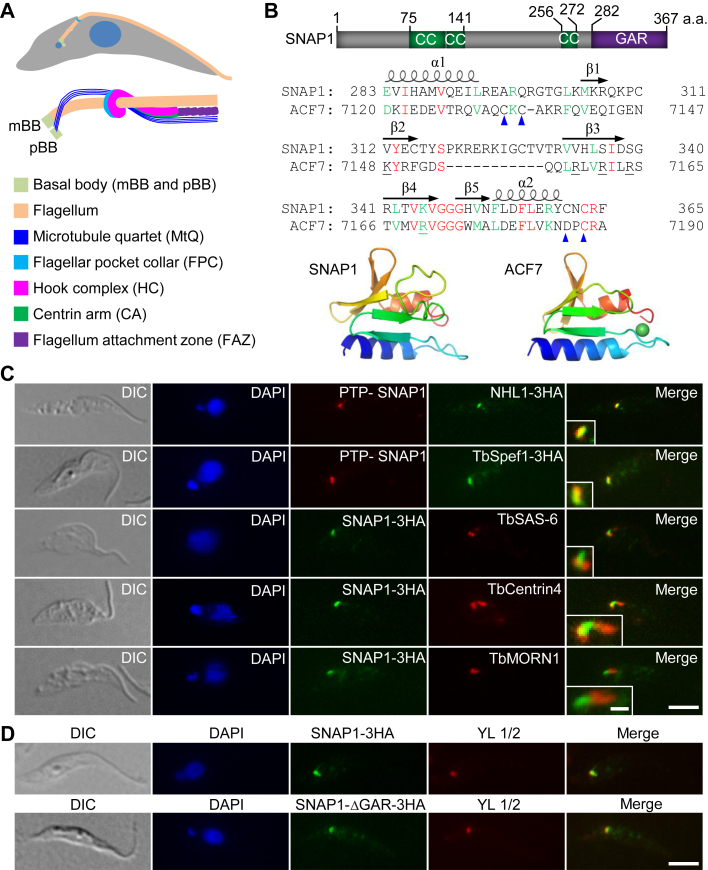
**SNAP1 localizes to the proximal portion of the microtubule quartet.***A*, schematic drawing of the cytoskeletal structures at the proximal portion of the *T. brucei* flagellum. The *top panel* shows a trypanosome cell with the flagellum (*orange*) and the flagellar pocket collar (*blue*) highlighted. The *bottom panel* illustrates the flagellum and its associated structures. mBB: mature basal body; pBB: pro-basal body. *B*, schematic illustration of the structural domains in SNAP1 and the sequence alignment and homology modeling of the GAR domains of SNAP1 and human ACF7. The template used for modeling is 5X57. CC: coiled coil. GAR: Gas2-related. *C*, subcellular localization of SNAP1 relative to other flagellum-associated cytoskeletal structures. NHL1, TbSAS-6, TbCentrin4, and TbMORN1 were used as markers for MtQ proximal region, basal body, basal body and centrin arm, and flagellar pocket collar, respectively. Scale bar: 5 μm. Scale bar in the zoom magnification image: 1 μm. *D*, subcellular localization of ectopically expressed SNAP1 and SNAP1-ΔGAR mutant. YL1/2 was used as the mBB marker. Scale bar: 5 μm.

After the duplicated organelles are fully segregated, trypanosome cells undergo an unusual mode of cytokinesis by assembling a cell division plane along the longitudinal cell axis between the two duplicated flagella (26), and the length of the newly formed flagellum and its associated FAZ appears to define the position of the cell division plane to ensure symmetrical cytokinesis for the production of two equally sized daughter cells (3, 14). Along the cell division plane, a cell division fold is formed through membrane invagination, and a cleavage furrow ingresses uni-directionally from the anterior cell tip of the new-flagellum daughter cell to the nascent posterior of the old-flagellum daughter cell, bisecting the bi-flagellated cell into two uni-flagellated daughter cells (26). Cytokinesis initiation and completion is controlled by several evolutionarily conserved regulators and numerous trypanosome-specific regulators that function in concert at the anterior tip of the new flagellum daughter cell and/or the cleavage furrow (27, 28, 29, 30, 31, 32, 33, 34, 35, 36, 37, 38). Two of these cytokinesis regulatory proteins, KLIF and FRW1, localize along the ingressing cleavage furrow and thus mark the cell division plane (32, 38), but they are not involved in determining the positioning of the cell division plane.

The essential role of the newly assembled flagellum and its associated FAZ in determining the position of the cell division plane suggests that the flagellum-associated cytoskeletal structures, including the MtQ, may play a role in controlling the positioning of the flagellum and the cell division plane. The physiological function of the MtQ remains elusive. Sixteen proteins have been localized to the proximal portion of the MtQ (39), but only two of them have been functionally characterized. The first characterized protein is the *T. brucei* homolog of human Sperm flagellar protein 1 (TbSpef1), which promotes microtubule bundling and is required for the duplication/segregation of multiple flagellum-associated cytoskeletal structures (40, 41). The second characterized protein is NHL1, which interacts with TbSpef1, depends on TbSpef1 for localization to the MtQ, and promotes basal body rotation and segregation, thereby facilitating the positioning of the flagellum and the cell division plane (25). However, the mechanisms by which TbSpef1 and NHL1 regulate the duplication and/or segregation of flagellum-associated cytoskeletal structures remain unclear. In this work, we identified a TbSpef1-and NHL1-associated protein named SNAP1, characterized its function in flagellum and cell division plane positioning, and investigated its functional interplay with TbSpef1 and NHL1. Our results revealed a critical role for SNAP1 in recruiting NHL1 to promote flagellum positioning and cell division plane placement through the regulation of basal body rotation and segregation.

## Results

### SNAP1 localizes to the proximal end of the microtubule quartet

Our recent characterization of the MtQ-localized protein NHL1 and its essential role in promoting flagellum inheritance and cell division plane positioning (25) prompted us to identify other MtQ-localized proteins that may cooperate with NHL1. Among the 159 epitope-tagged trypanosome essential proteins (4), a hypothetical protein encoded by Tb927.9.7090 was found to localize to the MtQ and associate with NHL1 and TbSpef1 (see below). We named this protein SNAP1 (TbSpef1-and NHL1-Associated Protein 1) and characterized its function in the procyclic form of *T. brucei*. Structural modeling using SWISS-MODEL (42) showed that SNAP1 contains a GAR (Gas2-related)-like motif at its C-terminus and three coiled-coil motifs in the middle region of the protein (Fig. 1*B*). The GAR domain is mainly found in Gas2-family and plakin-family proteins, and it is an α/β sandwich composed of five anti-parallel β-strands flanked by two α-helices (Fig. 1*B*). This domain is capable of binding microtubules, likely through the positively charged residues in the central β-sheet (Fig. 1*B*, residues underlined) (43). However, the GAR domain in the GAS2L3 protein was found to interact with the chromosomal passenger complex subunits Survivin and Borealin for the localization of GAS2L3 to the constriction zone during abscission (44), suggesting that the GAR domain also mediates protein-protein interaction. Using a tetrahedral coordination geometry, the GAR domain coordinates a zinc ion with the conserved Cys-Cys-Asp-Cys residues located in the α1-β1 loop and the C-terminal loop flanking the α2 helix (Fig. 1*B*, arrowheads) (45). However, four out of the five positively charged residues that mediate microtubule binding, and three out of the four residues that coordinate zinc binding are missing in the putative GAR domain of SNAP1 (Fig. 1*B*), raising the question of whether this putative GAR domain is able to bind microtubules. This domain in SNAP1 might be involved in protein-protein interaction, as is the case in GAS2L3.

We generated *T. brucei* cell lines expressing SNAP1 tagged with either a PTP epitope or a triple HA epitope from one of its endogenous loci and performed co-immunofluorescence microscopy with antibodies against the protein components of various flagellum-associated cytoskeletal structures to determine the precise location of SNAP1. Co-immunostaining of cells for PTP-SNAP1 and NHL1-3HA or TbSpef1-3HA, both of which localize to the proximal region of MtQ (25, 40), showed that SNAP1 co-localizes with both NHL1 and TbSpef1 (Fig. 1*C*). Further, co-immunostaining for SNAP1-3HA and the basal body cartwheel protein TbSAS-6 (5) showed that the proximal end of SNAP1 is located between the mature basal body and the pro-basal body (Fig. 1*C*). Co-immunostaining for SNAP1-3HA and TbCentrin4, which labels both the basal body and the centrin arm of the hook complex, showed that SNAP1 extends from the basal body to the centrin arm (Fig. 1*C*). Moreover, co-immunostaining for SNAP1-3HA and TbMORN1, which stains the fishhook-like structure of the hook complex (46, 47), showed that SNAP1 localizes to the region proximal to the fishhook-like structure (Fig. 1*C*). Finally, to test whether the putative GAR domain is required for SNAP1 localization, we ectopically expressed full-length SNAP1 and the GAR-deleted mutant form of SNAP1 in trypanosomes, and immunofluorescence microscopy showed that deletion of the putative GAR domain did not affect the localization of SNAP1 (Fig. 1*D*). Together, these results suggest that SNAP1 co-localizes with TbSpef1 and NHL1 at the proximal part of the MtQ between the basal body and the hook complex, independently of the putative GAR domain.

### SNAP1 knockdown impairs cytokinesis and causes misplacement of the cleavage furrow

**Figure 2:**
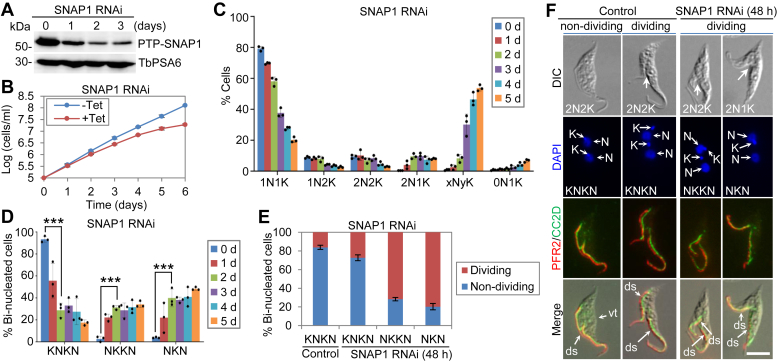
**Knockdown of SNAP1 impairs cytokinesis completion and cleavage furrow positioning.***A*, knockdown of SNAP1 by RNAi in procyclic trypanosomes. Endogenous PTP-SNAP1 was detected by anti-Protein A antibody. TbPSA6 served as a loading control. *B*, RNAi of SNAP1 caused growth defects. *C*, SNAP1 knockdown impaired cell division. Cells with different numbers of nucleus (N) and kinetoplast (K) were counted for each time point. 1N1K, one nucleus and one kinetoplast; 1N2K, one nucleus and two kinetoplasts; 2N2K, two nuclei and two kinetoplasts; 2N1K, two nuclei and one kinetoplast; xNyK, more than two nuclei and one or more kinetoplasts; 0N1K, zero nucleus and one kinetoplast. Error bars indicate S.D. (n = 3). *D*, SNAP1 knockdown inhibited kinetoplast segregation. Bi-nucleated cells with different configuration of the nucleus and the kinetoplast were counted for each time point. Error bars indicate S.D. (n = 3). ∗∗∗*p* < 0.001 (Chi-square test). *E*, SNAP1 knockdown disrupted cytokinesis completion. Shown is the quantitation of dividing and non-dividing cells before and after SNAP1 RNAi. 100 cells were counted for each time point. Error bars represent S.D. (n = 3). *F*, knockdown of SNAP1 caused mis-positioning of the new-flagellum daughter cell of dividing cells. Flagellum and the FAZ were immunostained with anti-PFR2 antibody and anti-CC2D antibody, respectively. The *white arrows* in the DIC channel indicate the cleavage furrow. N, nucleus; K, kinetoplast; ds, dorsal; vt, ventral. Scale bar: 5 μm.

To study the function of SNAP1, a cell line was generated that harbors an RNAi construct targeting the SNAP1 coding sequence in the procyclic form of *T. brucei*. Induction of RNAi caused a gradual reduction of the level of SNAP1, which was endogenously tagged with an N-terminal PTP tag, as detected by western blotting (Fig. 2*A*). This knockdown of SNAP1 slowed down cell proliferation (Fig. 2*B*). We next analyzed its effect on cell cycle progression by counting the cells with different numbers of nucleus (N) and kinetoplast (K), the cell’s mitochondrial DNA complex. This is based on the fact that trypanosome cells at different cell cycle stages contain different numbers of nucleus and kinetoplast. Cells at G1 and early S-phase contain one nucleus and one kinetoplast (1N1K), cells from late S-phase to metaphase contain one nucleus and two kinetoplasts (1N2K), and cells from anaphase to cytokinesis contain two nuclei and two kinetoplasts (2N2K). Aberrant cell types, such as the so-called zoid cells containing only a kinetoplast but not nucleus (0N1K), the cells containing two nuclei and one kinetoplast (2N1K), and the polyploid cells containing more than two nuclei and one or more kinetoplasts (xNxK or xNyK, x > 2, y ≥ 1), emerge when cytokinesis is defective in some cell cycle gene-deficient cell lines. The quantitation data showed that RNAi of SNAP1 resulted in an accumulation of bi-nucleated (2N2K and 2N1K) cells from 24 h and polyploid (xNyK) cells from 72 h (Fig. 2*C*), suggesting that SNAP1 knockdown caused defects in cytokinesis. The generation of 2N1K cells could be due to either aberrant cytokinesis of the 2N2K cells, which generated 2N1K cells and 0N1K cells, or inhibited segregation of the duplicated kinetoplasts. The emergence of 0N1K cells (Fig. 2*C*) suggests that aberrant cytokinesis of 2N2K cells occurred in some of the 2N2K cells, which generated some of the 2N1K cells.

In wild-type bi-nucleated trypanosome cells, the two nuclei (N) and the two kinetoplasts (K) are positioned as the KNKN configuration (viewed from the cell posterior toward the cell anterior). When kinetoplast duplication or segregation is defective in some RNAi mutant cells, the bi-nucleated cells have either the NKKN configuration or the NKN configuration. We observed inhibited kinetoplast segregation in bi-nucleated cells after SNAP1 RNAi, as the bi-nucleated cells with the NKKN configuration or NKN configuration were gradually increased to ∼31% and ∼40%, respectively, followed by a decrease of the cells with the KNKN configuration from ∼93% to ∼29% after RNAi for 48 h (Fig. 2*D*). Strikingly, after SNAP1 RNAi for 48 h, ∼72% of the bi-nucleated cells with the NKKN configuration, ∼80% of the bi-nucleated cells with the NKN configuration, and ∼27% of the bi-nucleated cells with the KNKN configuration were undergoing cytokinesis, whereas only ∼16% of the bi-nucleated cells with the KNKN configuration from the control cell population were undergoing cytokinesis (Fig. 2, *E* and *F*), suggesting that SNAP1 RNAi caused defects in cytokinesis completion.

Finally, the dividing bi-nucleated cells from the RNAi cell population appeared to possess a mis-positioned cleavage furrow, with the dorsal sides of both daughter cells facing the ingressing cleavage furrow (Fig. 2*F*), in contrast to the dividing bi-nucleated cells from the non-induced control population, in which the cleavage furrow was placed between the ventral side of the new-flagellum daughter cell and the dorsal side of the old-flagellum daughter cell (Fig. 2*F*). This observation suggests that SNAP1 knockdown impairs the placement of the cleavage furrow.

### SNAP1 is required for positioning the cell division plane

**Figure 3:**
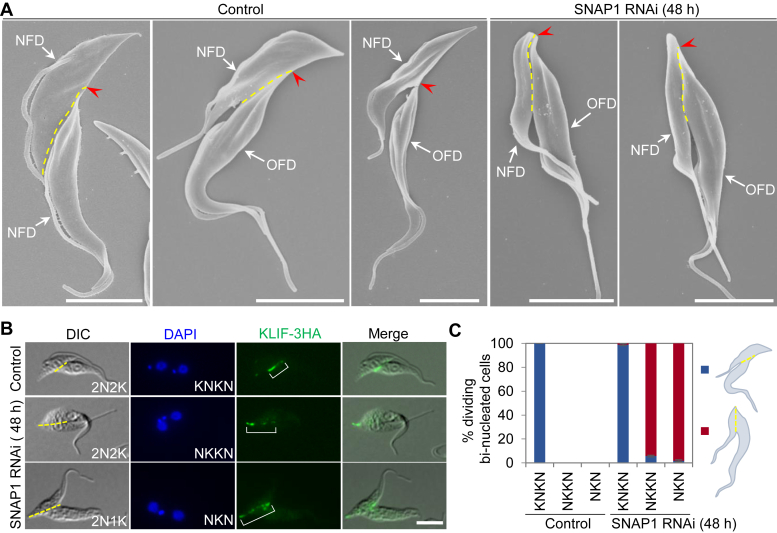
**Knockdown of SNAP1 disrupts cell division plane placement.***A*, visualization of the cell division plane in dividing cells by scanning electron microscopy. NFD, new-flagellum daughter; OFD, old-flagellum daughter. The *red open arrowhead* indicates the nascent posterior of the OFD cell, and the *yellow dashed line* marks the cell division plane. Scale bars: 5 μm. *B*, labeling of the cell division plane in dividing cells with KLIF as the cell division plane marker. KLIF-3HA was expressed from its endogenous locus. The bracket outlines the KLIF fluorescence signal. The *yellow dashed line* indicates the KLIF-marked cell division plane. Scale bar: 5 μm. *C*, quantitation of dividing cells with normal or mis-positioned cell division plane from control and SNAP1 RNAi cell population. 100 cells were counted for each time point and for each cell type. Error bars represent S.D. (n = 3). The cartoons on the right depict the cells with a normal (*blue*) or a mis-positioned (*red*) cell division plane, which is marked with a *yellow dashed line*.

One of the key cellular events prior to cleavage furrow ingression is the formation of a so-called cell division fold between the new and the old flagella (26), which marks the cell division plane from the anterior end of the new-flagellum daughter cell to the nascent posterior of the old-flagellum daughter cell (26, 33). Because SNAP1 RNAi impaired the placement of the cleavage furrow (Fig. 2*F*), we examined whether SNAP1 RNAi disrupted the positioning of the cell division plane. Prior to cytokinesis initiation and at early stages of cytokinesis, the non-induced control cells had a normally placed cell division plane, with the nascent posterior of the old-flagellum daughter cell located in the mid-portion of the new-flagellum daughter cell (Fig. 3*A*, left and middle panels of the control). At late stages of cytokinesis of the non-induced control cells, the nascent posterior of the old-flagellum daughter cell was still connected to the mid-portion of the new-flagellum daughter cell by a thin cytoplasm bridge (Fig. 3*A*, right panel of the control). However, in the SNAP1 RNAi-induced cells that were undergoing cytokinesis, the cell division plane apparently was mis-positioned, with the nascent posterior of the old-flagellum daughter cell located in close proximity to the existing posterior end of the new-flagellum daughter cell (Fig. 3*A*).

To further confirm the effect of SNAP1 RNAi on the positioning of the cell division plane, we immuno-labeled the cleavage furrow of dividing cells using the orphan kinesin KLIF as a marker of the cell division plane (32), and then quantitated the cells with normally or abnormally positioned cell division plane before and after SNAP1 RNAi induction (Fig. 3, *B* and *C*). All of the bi-nucleated cells with the KNKN configuration before and after SNAP1 RNAi had a normally positioned cell division plane (Fig. 3*C*). However, after SNAP1 RNAi induction, ∼94% of the bi-nucleated cells with the NKKN configuration and ∼98% of the bi-nucleated cells with the NKN configuration had a mis-positioned cell division plane (Fig. 3*C*). These results demonstrated that knockdown of SNAP1 impaired the placement of the cell division plane.

### SNAP1 is required for the positioning of the new flagellum and the elongation of the new FAZ

**Figure 4:**
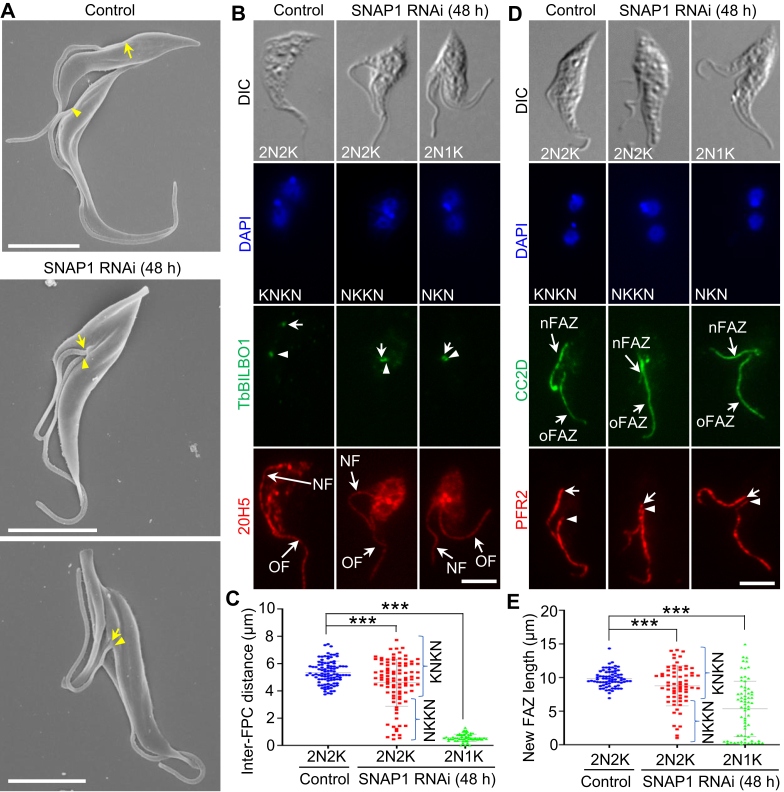
**SNAP1 is required for flagellum segregation and FAZ elongation.***A*, scanning electron microscopic analysis of control and SNAP1 RNAi cells, showing the position of the proximal base of the new and old flagella (*arrow* and *arrowhead*, respectively). Scale bars: 5 μm. *B*, effect of SNAP1 RNAi on the segregation of the flagellar pocket collar (FPC) and the flagellum. FPC and flagellum were immunostained with the anti-TbBILBO1 antibody and the 20H5 antibody, respectively. *Arrows* and *arrowheads* indicated the new FPC and the old FPC, respectively. NF, new flagellum; OF, old flagellum. Scale bar: 5 μm. *C*, measurement of the inter-FPC distance in control and SNAP1 RNAi cells. 100 cells for each cell type and each time point were used for measurement. Error bars indicate S.D. (n = 3). ∗∗∗*p* < 0.001 (Student’s *t* test). *D*, effect of SNAP1 RNAi on the elongation of the FAZ. Cells were immunostained with the anti-CC2D antibody and the anti-PFR2 antibody to label the FAZ and the flagellum, respectively. *Arrows* and *arrowheads* indicate the proximal base of the new flagellum and the old flagellum, respectively. nFAZ: new FAZ; oFAZ: old FAZ. Scale bar: 5 μm. *E*, measurement of the length of the new FAZ in control and SNAP1 RNAi cells. 100 cells for each cell type and each time point were used for measurement. Error bars indicate S.D. (n = 3). ∗∗∗*p* < 0.001 (Student’s *t* test).

The mis-positioning of the cell division plane caused by SNAP1 RNAi suggests that the positioning of the new flagellum was likely impaired, as it was previously demonstrated that the newly assembled flagellum determines the size of the new-flagellum daughter cell by defining the cell division plane in *T. brucei* (3). To test this possibility, we first used scanning electron microscopy to visualize the position of the flagellum in control and SNAP1 RNAi cells. The results showed that the two flagella of the bi-flagellated cells appeared to exit the cell body from a single flagellar pocket after SNAP1 RNAi, in contrast to that in the non-induced control cells (Fig. 4*A*), suggesting defective positioning and segregation of the newly assembled flagellum. Further, we performed immunofluorescence microscopy to label the flagellar pocket collar with the anti-TbBILBO1 antibody, which detects the FPC protein TbBILBO1 (22), and the flagellum with the 20H5 antibody, which detects the centrin proteins in the flagellum (48, 49), the basal body, and the centrin arm (10, 50, 51, 52). We then measured the distance between the two flagellar pocket collars in the bi-nucleated cells before and after SNAP1 RNAi induction for a quantitative assessment of the effect on flagellum segregation by SNAP1 RNAi. The results showed that in all of the bi-nucleated cells with the NKKN configuration or the NKN configuration after SNAP1 RNAi, the two flagellar pocket collars were closely associated with each other, in contrast to the well separated flagellar pocket collars in the bi-nucleated cells with the KNKN configuration from the control (Fig. 4, *B* and *C*). It should be noted that the bi-nucleated cells with the KNKN configuration after SNAP1 RNAi still possessed two well separated flagellar pocket collars (Fig. 4*C*); these cells must have already segregated the flagella prior to the induction of SNAP1 RNAi. Nonetheless, these results further confirmed that SNAP1 RNAi impaired flagellum positioning.

We observed that the dividing bi-nucleated cells with the NKKN configuration or the NKN configuration after SNAP1 RNAi appeared to contain a long, unattached new flagellum (Fig. 4, *A* and *B*), which suggests that the defective positioning of the new flagellum might have disrupted the elongation of the new FAZ. To test this possibility, we co-immunostained the cells with the anti-CC2D antibody and the anti-PFR2 antibody to label the FAZ filament and the flagellum, respectively, and then measured the length of the new FAZ filament (Fig. 4, *D* and *E*). The results showed that the length of the new FAZ in the bi-nucleated cells was significantly reduced after SNAP1 RNAi (Fig. 4, *D* and *E*). Additionally, anti-PFR2 immunostaining further confirmed the inhibited segregation of the new flagellum from the old flagellum (Fig. 4*D*). Together, these results demonstrated that SNAP1 is required for the elongation of the new FAZ.

### Knockdown of SNAP1 disrupts basal body rotation and segregation

**Figure 5:**
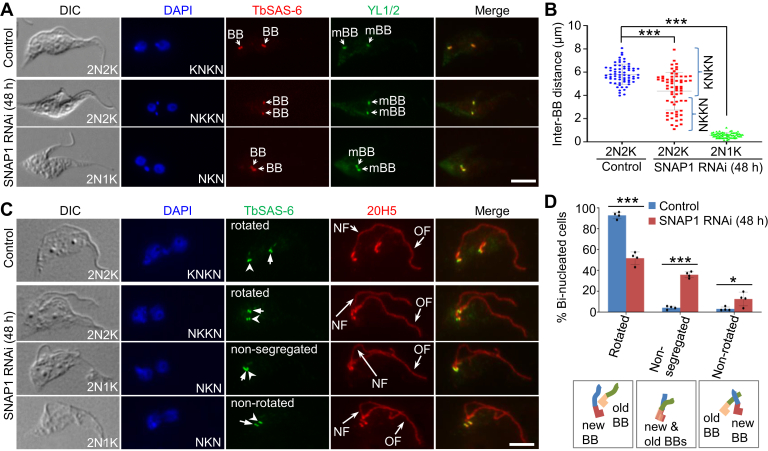
**SNAP1 is required for basal body rotation and segregation.***A*, effect of SNAP1 RNAi on basal body segregation. Cells were immunostained with the anti-TbSAS-6 antibody and the YL1/2 antibody to label the basal body (BB, including both the pro-basal body and the mature basal body) and the mature basal body (mBB), respectively. Scale bar: 5 μm. *B*, measurement of the inter-basal body distance in control and SNAP1 RNAi cells. 100 cells for each cell type and each time point were used for measurement. Error bars indicate S.D. (n = 3). ∗∗∗*p* < 0.001 (Student’s *t* test). *C*, effect of SNAP1 knockdown on basal body rotation. Cells were immunostained with the anti-TbSAS-6 antibody to label the pBB-mBB pair and the 20H5 antibody to label the basal body and its connected flagellum. *Arrowheads* and *arrows* indicate the new and the old pBB-mBB basal body pairs, respectively. NF, new flagellum; OF, old flagellum. Scale bar: 5 μm. *D*, quantitation of bi-nucleated cells with rotated, non-segregated, and non-rotated basal body pairs before and after SNAP1 RNAi. 100 cells for each time point were counted. Error bars indicate S.D. (n = 3). ∗*p* < 0.05; ∗∗∗*p* < 0.001 (Chi-square test).

During the early S phase of the cell cycle in *T. brucei*, the pBB develops to become an mBB, and a new pBB is then assembled next to each of the two mBBs, forming two pairs of pBB–mBB, with the newly assembled pBB–mBB pair located at the anterior side of the old pBB–mBB pair (8). A new flagellum is nucleated from the mBB of the newly assembled pBB–mBB pair, and this new flagellum is also located at the anterior side of the old flagellum. Subsequently, the new pBB–mBB pair and its associated new flagellum make a rotational movement toward the posterior side of the old pBB–mBB pair. The new flagellum then elongates and extends toward the cell anterior, and the new pBB–mPP further moves toward the cell posterior in accordance with the elongation of the new flagellum (8). We examined whether basal body segregation and/or rotation was affected in SNAP1 RNAi cells. Immunofluorescence microscopy was performed with the antibody against the basal body cartwheel protein TbSAS-6, which is located in both the pBB and the mBB (5), and the YL1/2 antibody, which labels the mBB (53, 54), and the inter-basal body distance in the bi-nucleated cells was measured and compared between the control and SNAP1 RNAi cells. The results showed a significant reduction in the inter-basal body distance in the bi-nucleated cells (2N2K and 2N1K) after depletion of SNAP1 by RNAi (Fig. 5, *A* and *B*), demonstrating that RNAi of SNAP1 inhibited basal body segregation.

We also wondered whether SNAP1 RNAi might affect basal body rotation. To test this possibility, we performed co-immunofluorescence microscopy using the anti-TbSAS-6 antibody to stain the basal body and the pan-centrin antibody 20H5 to label the basal body and its associated flagellum. By doing so, it allows us to distinguish between the new pBB-mBB pair and the old pBB-mBB pair, based on their associated new and old flagella, which can be easily distinguished (Fig. 5*C*). We again focused on the bi-nucleated cells, as all of them from the non-induced control contained a fully rotated new pBB–mBB pair (Fig. 5, *C* and *D*). In the bi-nucleated cells from the SNAP1 RNAi population, however, rotation of the new pBB-mBB pair only occurred in ∼52% of the bi-nucleated cells, and in ∼14% of the bi-nucleated cells the new pBB-mBB pair was not rotated (Fig. 5, *C* and *D*). Of the remaining bi-nucleated cells (∼36%), the new and the old pBB–mBB pairs were too closely associated to tell whether basal body rotation had occurred or not (Fig. 5, *C* and *D*); thus, these cells were classified as cells containing non-segregated pBB–mBB pairs. Altogether, these results demonstrated that SNAP1 is required for basal body rotation and segregation.

### SNAP1 associates with NHL1 and is required for NHL1 localization to the MtQ

**Figure 6:**
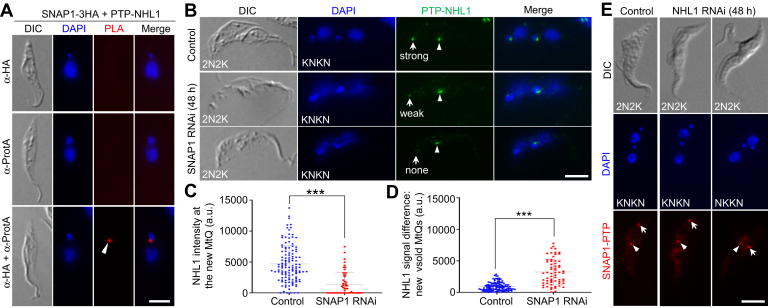
**SNAP1 associates with NHL1 and is required for NHL1 localization to the MtQ.***A*, PLA to test the *in vivo* interaction between SNAP1 and NHL1. Cells co-expressing endogenously C-terminally 3HA-tagged SNAP1 and N-terminally PTP-tagged NHL1 were used for PLA assay. Arrowhead indicates the positive signal at the MtQ proximal region. Scale bar: 5 μm. *B*, effect of SNAP1 knockdown on NHL1 localization. Endogenous PTP-NHL1 was immunostained with the anti-Protein A antibody and the FITC-conjugated anti-rabbit IgG. *Arrows* and *arrowheads* indicate the NHL1 signal at the new MtQ and the old MtQ, respectively. Scale bar: 5 μm. *C*, quantitation of NHL1 fluorescence signal intensity at the new MtQ in bi-nucleated control cells and SNAP1 RNAi cells. Error bars indicate the S.D. (n = 3). ∗∗∗*p* < 0.001 (Student’s *t* test). *D*, difference of the NHL1 fluorescence signal intensity between the new MtQ and the old MtQ. Error bars indicate S.D. (n = 3). ∗∗∗*p* < 0.001 (Student’s *t* test). *E*, effect of NHL1 knockdown on SNAP1 localization. Endogenous SNAP1-PTP was immunostained with the anti-Protein A antibody and the Cy3-conjugated anti-rabbit IgG. *Arrows* and *arrowheads* indicate SNAP1-PTP signal at the new MtQ and the old MtQ, respectively. Scale bar: 5 μm.

The previous finding that NHL1 and TbSpef1 form a complex (25) and the co-localization of SNAP1 with NHL1 and TbSpef1 to the proximal portion of the MtQ (Fig. 1*C*) prompted us to investigate whether SNAP1 is a subunit of a tri-protein complex. We carried out proximity ligation assay, which detects *in situ* protein-protein interaction in cells (55), and the results showed that SNAP1 and NHL1 interacted *in vivo* at the proximal region of the MtQ (Figs. 6*A* and S1). We next investigated whether knockdown of SNAP1 might affect NHL1 localization or vice versa. Immunofluorescence microscopy showed that the intensity of NHL1 fluorescence signal at the new MtQ, but not the old MtQ, of the bi-nucleated cells was significantly reduced after SNAP1 RNAi (Fig. 6, *B*–*D*). Conversely, when NHL1 was knocked down, the intensity of SNAP1 fluorescence signal at the new MtQ of the bi-nucleated cells was not affected (Fig. 6*E*). These results suggest that SNAP1 is required for recruitment of NHL1 to the new MtQ, but SNAP1 localization to the MtQ is independent of NHL1.

### SNAP1 associates with TbSpef1 and depends on TbSpef1 for localization to the MtQ

**Figure 7:**
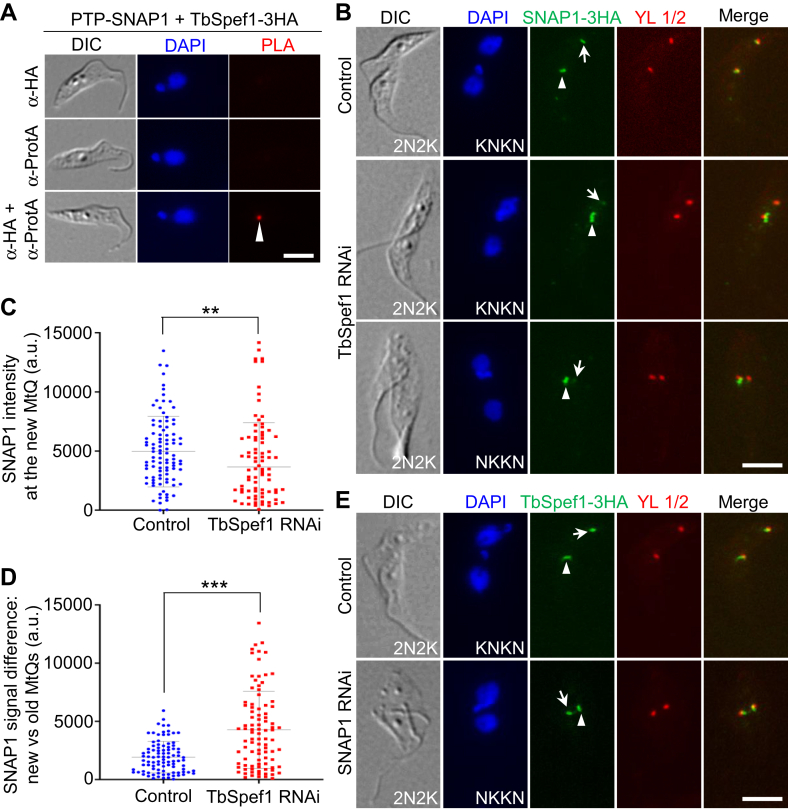
**SNAP1 associates with TbSpef1 and depends on TbSpef1 for localization to the MtQ.***A*, *in vivo* interaction between SNAP1 and TbSpef1 detected by PLA. Cells co-expressing endogenously N-terminally PTP-tagged SNAP1 and C-terminally 3HA-tagged TbSpef1 were used for PLA assay. Arrowhead indicates the positive signal at the MtQ proximal region. Scale bar: 5 μm. *B*, effect of TbSpef1 knockdown on SNAP1 localization. Endogenous SNAP1-3HA was immunostained with the FITC-conjugated anti-HA antibody. *Arrows* and *arrowheads* indicate the SNAP1 signal at the new MtQ and the old MtQ, respectively. Scale bar: 5 μm. *C*, quantitation of SNAP1 fluorescence signal intensity at the new MtQ and the old MtQ in bi-nucleated control cells and TbSpef1 RNAi cells. Error bars indicate the S.D. (n = 3). ∗∗*p* < 0.01 (Student’s *t* test). *D*, difference of the SNAP1 fluorescence signal intensity between the new MtQ and the old MtQ. Error bars indicate S.D. (n = 3). ∗∗∗*p* < 0.001 (Student’s *t* test). *E*, effect of SNAP1 knockdown on TbSpef1 localization. Endogenous TbSpef1-3HA was immunostained with the FITC-conjugated anti-HA antibody. *Arrows* and *arrowheads* indicate TbSpef1-3HA signal at the new MtQ and the old MtQ, respectively. Scale bar: 5 μm. YL1/2 in panels *D* and *E* was used to label the basal body for determining the relative position of the MtQ.

Because NHL1 interacts with TbSpef1 (25), and SNAP1 interacts with NHL1 (Figs. 6*A* and S1), we investigated whether SNAP1 also interacts with TbSpef1. Using proximity ligation assay, we showed that SNAP1 and TbSpef1 interacted *in vivo* in trypanosome cells (Figs. 7*A* and S1). Therefore, SNAP1, NHL1, and TbSpef1 appear to form a tri-protein complex at the proximal end of the MtQ. Among the three proteins, SNAP1 (Fig. 6) and TbSpef1 (25) are required for recruitment of NHL1 to the MtQ, but the functional relationship between SNAP1 and TbSpef1 is unclear. Since TbSpef1, but not SNAP1 (data not shown), can bind microtubules, we first tested whether TbSpef1 knockdown affects SNAP1 localization. Immunofluorescence microscopy was performed to examine the localization of SNAP1, which was endogenously tagged with a triple HA epitope, in TbSpef1 RNAi cells, and the results showed that the intensity of the SNAP1 fluorescence signal at the new MtQ, but not the old MtQ, of the bi-nucleated cells was significantly reduced (Fig. 7, *B*–*D*). Conversely, knockdown of SNAP1, however, did not affect TbSpef1 localization to the new MtQ (Fig. 7*E*). These results suggest that SNAP1 localization to the MtQ depends on TbSpef1, but not vice versa.

### The SNAP1-NHL1-TbSpef1 tri-protein complex and their structural motifs

**Figure 8:**
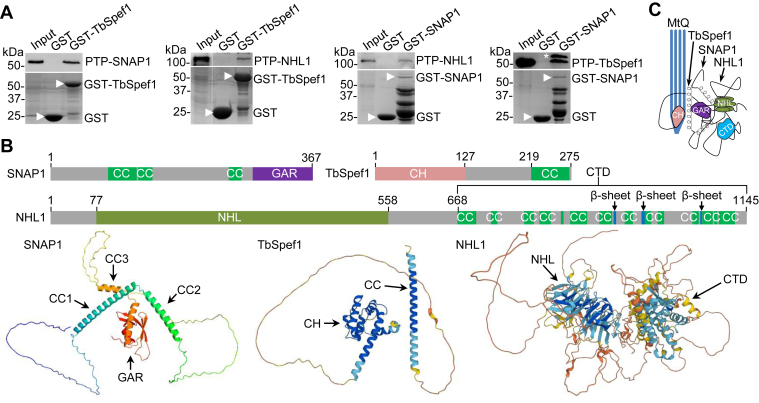
**Interactions and structural modeling of SNAP1, NHL1, and TbSpef1.***A*, GST pull-down to test the *in vitro* interactions among SNAP1, NHL1 and TbSpef1. Recombinant GST-TbSpef1 and GST-SNAP1 were used to pull down PTP-tagged SNAP1, NHL1, or TbSpef1 from *T. brucei* cell lysate. The *asterisk* indicates a non-specific band detected by the anti-Protein A antibody. GST and GST-fusion proteins are indicated by white *arrowheads*. *B*, schematic illustration of the structural domains of SNAP1, TbSpef1, and NHL1, and structures of SNAP1, TbSpef1, and NHL1 predicted by AlphaFold. CC, coiled-coil; GAR, Gas2-related; CH, calponin homology; NHL, Ncl1, HT2A, and Lin41; CTD, C-terminal domain. *C*, model of SNAP1, TbSpef1, and NHL1 interaction and association with the MtQ.

We sought to confirm the interaction among the three proteins, SNAP1, NHL1, and TbSpef1, by *in vitro* GST pull-down experiments. Recombinant GST-fused TbSpef1 had some truncation products, whereas GST-SNAP1 had many abundant truncation products (Fig. 8*A*), which were due to protein degradation in bacteria. Recombinant GST-fused NHL1, GST-fused NHL1 N-terminal NHL domain, and GST-fused C-terminal domain (CTD) were all insoluble in bacteria, and we were not able to purify these recombinant proteins despite numerous attempts under different conditions. Therefore, GST pull-down was performed with GST-TbSpef1 and GST-SNAP1 only. The results showed that TbSpef1 was able to bring down PTP-tagged SNAP1 and NHL1 from *T. brucei* cell lysate, and SNAP1 was able to pull down PTP-tagged NHL1 and TbSpef1 from *T. brucei* cell lysate (Fig. 8*A*), demonstrating that these three proteins form a complex and confirming the results obtained with proximity ligation assays (Figs. 6*A* and 7A).

Based on the prediction by AlphaFold (56, 57), SNAP1 contains three coiled-coil motifs and a C-terminal GAR domain (Fig. 8*B*), consistent with the prediction by SWISS MODEL (Fig. 1*B*). AlphaFold also predicted that TbSpef1 contains an N-terminal Calponin homology domain and a C-terminal coiled-coil motif (Fig. 8*B*), similar to the structure of the human Spef1 ortholog (58). The Calponin domain of human Spef1 binds and bundles microtubule, and the coiled-coil motif is required for dimerization (58). AlphaFold predicted that NHL1 contains an N-terminal NHL-like motif (Fig. 8*B*), a six-blade β-propeller structure involved in protein-protein interaction, as predicted by SWISS-MODEL previously (25). Intriguingly, AlphaFold predicted an unusual C-terminal domain composed of multiple coiled-coil motifs and β-sheets (Fig. 8*B*), which has not been found in any other proteins in any organisms and has no known functions. Together, we propose that this tri-protein complex associates with the proximal end of the MtQ, with TbSpef1 binding directly to microtubules and NHL1 and SNAP1 interacting with TbSpef1 for association with the MtQ (Fig. 8*C*).

## Discussion

In this report, we characterized a new MtQ-localized protein named SNAP1, which plays an essential role in promoting basal body rotation and segregation in the procyclic form of *T. brucei*. Among the dozen MtQ-localized proteins in *T. brucei*, only two of them, TbSpef1 and NHL1, have been previously characterized, which appear to play distinct roles despite that they form a complex (25, 40). TbSpef1 possesses microtubule bundling activity (41), likely through its N-terminal Calponin domain because its human ortholog (HsSpef1) uses this domain for microtubule binding and bundling (58), and plays a role in regulating the duplication of several flagellum-associated cytoskeletal structures (40). NHL1 interacts with TbSpef1 *in vivo* at the proximal end of the MtQ, and plays a role in promoting basal body rotation and segregation, thereby facilitating flagellum positioning and ensuring correct placement of the cleavage furrow (25). Given the lack of any putative microtubule-binding motifs in NHL1 and the dependence on TbSpef1 for NHL1 localization to the MtQ (25), it suggests that NHL1 associates with the MtQ indirectly through interaction with TbSpef1, which may bind the MtQ directly through its Calponin domain, as is the case of the human Spef1 protein (58). The newly identified MtQ-associated protein SNAP1 contains a putative microtubule-binding domain (the GAR domain), but this domain lacks several key residues required for microtubule binding (Fig. 1*B*), and the recombinant eYFP-SNAP1 expressed and purified from *E. coli* does not bind microtubules *in vitro* (data not shown), suggesting the lack of microtubule-binding capability for SNAP1. Thus, like NHL1, SNAP1 may also associate with the MtQ indirectly through the interaction with TbSpef1.

We have identified a tri-protein complex composed of TbSpef1, NHL1, and SNAP1, which is located at the proximal end of the MtQ. Both NHL1 and SNAP1 depend on TbSpef1 for localization to the MtQ, but not vice versa (Fig. 7 and (25)), and NHL1 additionally depends on SNAP1 for localization to the MtQ, but not vice versa (Fig. 6). Therefore, it appears that SNAP1 resides in the middle of this tri-protein complex, with TbSpef1 binding to the MtQ directly and bundling microtubules at the proximal end of the MtQ (Fig. 8*C*). The four microtubules of the MtQ originate from the region between the mBB and the pBB, and this tri-protein complex localize to the proximal end of the MtQ (Fig. 1). Given that TbSpef1 has microtubule bundling activity (41), we postulate that TbSpef1 promotes the bundling of the four microtubules at the proximal end of the MtQ (Fig. 8*C*). However, the mechanistic roles of NHL1 and SNAP1 remain unknown. Because the structural motifs in the two proteins are involved in protein-protein interactions, we speculate that the two proteins either help to recruit additional proteins to the proximal end of the MtQ or modulate the microtubule-bundling activity of TbSpef1 to facilitate microtubule bundling at the proximal end of the MtQ.

Knockdown of SNAP1 impaired the positioning of the newly assembled flagellum and reduced the length of its associated FAZ (Fig. 4), without affecting the length of the new flagellum (data not shown). Previously, it has been demonstrated that the positioning of the newly assembled flagellum depends on the faithful segregation of multiple flagellum-associated cytoskeletal structures, including the hook complex, the basal body, and the FAZ (5, 13, 34). It was also suggested that the new flagellum contributes to its own positioning because the inhibition of new flagellum assembly or new FAZ elongation restricts basal body migration toward the cell posterior (59). However, although knockdown of SNAP1 inhibited basal body segregation (Fig. 5) and impaired FAZ elongation (Fig. 4, *D* and *E*), there is no linear correlation between the inter-basal body distance and the new FAZ length, particularly in the 2N1K cells, of which ∼62% have a shorter new FAZ but all (100%) have a very short inter-basal body distance (Figs. 4*E* and 5B). Therefore, the inhibited basal body segregation by SNAP1 RNAi is unlikely attributed to the defective FAZ elongation. Additionally, given that flagellum assembly was unaffected by SNAP1 RNAi (Fig. 4), these results suggest that it was the inhibited basal body segregation that contributed to the impaired flagellum positioning in SNAP1 RNAi cells.

Depletion of SNAP1 caused the mis-positioning of the cell division plane in dividing trypanosome cells, resulting in the placement of the nascent posterior in close proximity to the existing posterior, instead of at the mid-portion of the ventral side of the new-flagellum daughter cell (Fig. 3). Previously, it was suggested that the length of the new flagellum and its associated FAZ defines the cell division plane in *T. brucei* (3, 14). Since SNAP1 RNAi also reduced the new FAZ length in ∼21% of the 2N2K cells and ∼62% of the 2N1K cells (Fig. 4, *D* and *E*), it raised the question of whether this impaired elongation of the new FAZ contributed to the mis-placement of the cell division plane. However, ∼94% of the 2N2K cells with an NKKN configuration (∼38% of all the 2N2K cells) and ∼98% of the 2N1K cells have a mis-positioned cell division plane (Fig. 3*C*), which argues against the contribution of the impaired new FAZ elongation to the mis-placement of the cell division plane. Instead, there appears to be a positive correlation between the cells with a mis-positioned cell division plane and the cells with a reduced inter-basal body distance (Figs. 3*C* and 5B), because almost all of the 2N2K cells with an NKKN configuration and all of the 2N1K cells have a reduced inter-basal body distance (Fig. 5*B*). Since the inhibition in basal body segregation contributes to the mis-positioning of the new flagellum (see above), we postulate that the impaired flagellum positioning contributes to the mis-placement of the cell division plane in SNAP1 RNAi cells.

Because SNAP1 localizes to the proximal end of the MtQ next to the basal body (Fig. 1), it suggests that the primary function of SNAP1 is to regulate certain cellular activities at this specific subcellular location. Knockdown of SNAP1 does not appear to affect the attachment of MtQ to the basal body, based on the unchanged localization of TbSpef1 in SNAP1 RNAi cells (Fig. 7*E*). Knockdown of the basal body protein TbSAF1, a TbSpef1-associated protein in *T. brucei*, disrupted the connection of the MtQ to the basal body (41). It suggests that TbSAF1 mediates the connection between the MtQ and the basal body, which appears to be essential for the positioning of the flagellum, likely through promoting basal body rotation and segregation (41). Although the mechanistic role of SNAP1 remains unclear, we postulate that SNAP1, by forming a complex with TbSpef1 and NHL1 at the proximal end of the MtQ, plays a role by strengthening the MtQ-basal body connection to facilitate the rotation and migration of the newly matured pro-basal body toward the posterior portion of the cell. In the absence of SNAP1, the connection between the MtQ and the basal body is weakened; thus, the pushing force generated through the extension of the MtQ becomes too weak to push the newly matured pro-basal body to rotate and migrate toward the cell posterior, thereby inhibiting basal body rotation and segregation. Since RNAi of NHL1 causes similar defects as SNAP1 RNAi and SNAP1 localization to the MtQ is independent on NHL1 (Fig. 6*E*), it suggests that strengthening the MtQ-basal body connection requires both SNAP1 and NHL1.

In summary, we have discovered a tri-protein complex that localizes to the proximal end of the MtQ to facilitate the rotation and migration of the newly matured pro-basal body toward the cell posterior, thereby promoting flagellum positioning and cell division plane placement for cell division in *T. brucei*. This tri-protein complex may bundle the four microtubules at the proximal end of the MtQ through TbSpef1’s microtubule bundling activity, and may connect to the basal body through TbSpef1’s association with the basal body protein TbSAF1 (41) to promote the rotation of the newly matured pro-basal body for the positioning of the newly assembled flagellum and the placement of the cell division plane for a successful cytokinesis.

## Experimental procedures

### Trypanosome cell culture and RNA interference

The procyclic form of *T. brucei* 29 to 13 strain (60) was cultured at 27 °C in the SDM-79 medium containing 10% heat-inactivated fetal bovine serum (Atlanta Biologicals, Inc), 15 μg/ml G418, and 50 μg/ml hygromycin. To generate a SNAP1 RNAi cell line, a 707-bp DNA fragment (nucleotides 293–1000) from the coding region of the *SNAP1* gene was cloned into the pZJM vector (61), and the resulting plasmid, pZJM-SNAP1, was linearized with NotI digestion and used to transfect the 29 to 13 strain by electroporation. Transfectants were selected by incubating with 2.5 μg/ml phleomycin and further cloned by limiting dilution in a 96-well plate containing SDM-79 medium supplemented with 20% heat-inactivated fetal bovine serum and appropriate antibiotics. The NHL1 RNAi cell line and the TbSpef1 RNAi cell line were generated previously (25). RNAi was induced with 1.0 μg/ml tetracycline, and cell growth was monitored daily by counting the number of cells before and after RNAi induction.

### Endogenous epitope tagging of proteins

Endogenous tagging of SNAP1, NHL1, and TbSpef1 with a C-terminal triple HA epitope or with an N-terminal PTP epitope was carried out using the PCR-based one-step epitope tagging method (62). PCR products were purified and electroporated into the *T. brucei* Lister427 strain, the SNAP1 RNAi cell line, the NHL1 RNAi cell line, or the TbSpef1 RNAi cell line. Successful transfectants were selected with appropriate antibiotics and cloned by limiting dilution in a 96-well plate containing SDM-79 medium supplemented with 20% heat-inactivated FBS and appropriate antibiotics.

For co-tagging of SNAP1 with NHL1 or TbSpef1 in the same cell line for co-localization and proximity ligation assay, SNAP1 was endogenously tagged with an N-terminal PTP epitope or with a C-terminal triple HA epitope, whereas NHL1 and TbSpef1 were each tagged with a C-terminal triple HA epitope or an N-terminal PTP epitope in the *T. brucei* Lister427 strain using the PCR-based one-step epitope tagging method. Successful transfectants were selected with appropriate antibiotics, and were further cloned by limiting dilution as described above.

### Immunofluorescence microscopy

*T. brucei* cells were washed with PBS, settled on coverslips, and fixed with methanol at −20 °C. To prepare *T. brucei* cytoskeletons, cells settled on coverslips were treated with 1% Nonidet-P40 in PEME buffer (100 mM PIPES, pH 6.9, 2 mM EGTA, 1 mM MgSO_4_, and 0.1 mM EDTA) for 1 s at room temperature and fixed with cold methanol at −20 °C. Intact cells or cytoskeletons were rehydrated with PBS, incubated with blocking buffer (3% BSA in PBS) for 30 min at room temperature, and then incubated with a primary antibody for 60 min at room temperature. The following primary antibodies were used: fluorescein isothiocyanate (FITC)-conjugated anti-HA monoclonal antibody (Sigma-Aldrich; 1:400 dilution), anti-PFR2 polyclonal antibody (clone L8C4, 1:40 dilution) (63), anti-TbMORN1 polyclonal antibody (46) (1:400 dilution), anti-TbCentrin4/LdCentrin1 polyclonal antibody (50) (1:1000 dilution), anti-TbBILBO1 polyclonal antibody (22) (1:1000 dilution), anti-CC2D polyclonal antibody (14) (1:1000 dilution), anti-TbSAS-6 polyclonal antibody (5) (1:1000 dilution), pan-centrin 20H5 monoclonal antibody (64) (1:400 dilution), YL1/2 monoclonal antibody (65) (1:1000 dilution), and anti-Protein A polyclonal antibody (Sigma-Aldrich, 1:400 dilution). Cells or cytoskeletons on the coverslip were washed three times with PBS, and then incubated with appropriate secondary antibodies for 60 min at room temperature. The following secondary antibodies were used: FITC-conjugated anti-rabbit IgG, Alexa Fluor594-conjugated anti-rat IgG, Cy3-conjugated anti-rabbit IgG, and Cy3-conjugated anti-mouse IgG. Cells or cytoskeletons were washed three times with PBS, mounted in VectaShield mounting medium (Vector Lab), and observed with an inverted fluorescence microscope (Olympus IX71) equipped with a cooled CCD camera and a PlanApo N 60X1.42 NA oil lens. Images were acquired with the Slidebook software.

### Proximity ligation assay

Proximity ligation assay (PLA) was performed with the Duolink *In Situ* PLA Detection kit, following manufacturer’s instructions (Cat#: DUO92008, Sigma-Aldrich). *T. brucei* cells co-expressing SNAP1-3HA and PTP-NHL1 or co-expressing PTP-SNAP1 and TbSpef1-3HA were settled on coverslips and fixed in cold methanol (−20 °C). Cells were incubated with Duolink blocking solution and then incubated with anti-HA and anti-Protein A antibodies. Cells on the coverslip were washed with Buffer A, and then probed with Duolink *In Situ* PLA Probe anti-Mouse MINUS (#DUO92004, Sigma Aldrich) and Duolink *In Situ* PLA Probe anti-Rabbit PLUS (#DUO92002, Sigma Aldrich). Subsequently, cells were washed with Buffer A, incubated with the ligation solution, and then incubated with the amplification solution in a humidity chamber at 37 °C for 100 min. Finally, cells were washed with Buffer B, mounted in Duolink *In Situ* Mounting Medium, and examined under an inverted fluorescence microscope (Olympus IX71). For negative controls, the *T. brucei* strain 29 to 13, the 29 to 13 strain expressing only PTP-tagged TbSpef1, and the 29 to 13 strain expressing only the 3HA-tagged TbSNAP1 were included in the PLA experiments, and the results were presented in Fig. S1.

### GST pull-down and Western blotting

The coding sequence of TbSpef1 and SNAP1 was each cloned into the pGEX-4T-3 vector (Clontech) for expression of recombinant GST-fused TbSpef1 and SNAP1 in bacteria. The resulting plasmids, pGEX-TbSpef1 and pGEX-SNAP1, and the empty vector were used to transform the *E. coli* BL21 strain. Expression of recombinant GST-TbSpef1, GST-SNAP1, or GST alone was induced with 0.1 mM IPTG at room temperature for 16 h. Bacteria cells expressing recombinant proteins were lysed in 1.0 ml lysis buffer (0.1% Triton X-100 in PBS) by sonication (5 s on and 10 s off for a total of 5 min), and cell lysate was cleared by centrifugation at the highest speed (20,267*g*) in a microcentrifuge at 4 °C. The cleared cell lysate was then incubated with 25 μl glutathione sepharose 4B (GE HealthCare) and bound proteins were washed thoroughly with 0.1% Triton X-100 in PBS.

The trypanosome cell lysate was prepared from ∼10^7^ cells by sonicating cells in 1 ml lysis buffer (25 mM Tris-HCl, pH 7.6, 150 mM NaCl, 1 mM DTT, 1% NP-40, and protease inhibitor cocktail), and cell lysate was cleared by centrifugation at the highest speed (20,267*g*) in a microcentrifuge at 4 °C. 50 μl out of the 1.0 ml cell lysate was saved as the input sample, and the remaining cell lysate was split into two fractions (475 μl each). The trypanosome cell lysate was incubated with GST-TbSpef1, GST-SNAP1, and GST bound to the glutathione sepharose 4B beads, respectively, for 1 h at 4 °C with gentle rotation.

The beads were then washed five times with the trypanosome cell lysis buffer, and bound proteins and their interacting partner proteins were eluted with 30 μl of the 1× SDS-PAGE sampling buffer. Eluted proteins were separated by SDS-PAGE and analyzed by western blotting using the anti-Protein A polyclonal antibody (Sigma-Aldrich, 1:1000 dilution) to detect PTP-tagged proteins. Recombinant GST-fused proteins and GST were stained with Coomassie brilliant blue.

### Scanning electron microscopy

Scanning electron microscopy was carried out as previously described (33). Briefly, trypanosome cells were fixed directly in the flask by adding 2.5% (v/v) glutaraldehyde into the culture medium at room temperature for 2 h. The fixed cells were collected by centrifugation at 750*g* for 10 min, washed twice with PBS by centrifugation, settled on coverslips for 30 min, and then dehydrated with alcohol (30%, 50%, 70%, 90%, and 100%) for 5 min each. After critical point drying, cells on the coverslip were coated with a 5-nm metal film (Pt:Pd 80:20, Ted Pella Inc). Cells were imaged with Nova NanoSEM 230 (FEI). The parameters used were 5 mm for the scanning work distance and 8 kV for the accelerating high voltage.

### Homology-based structural modeling of proteins by SWISS-MODEL and AlphaFold

Analysis of SNAP1, NHL1, and TbSpef1 structural domains was carried out using the SWISS-MODEL software (https://swissmodel.expasy.org). The template used for modeling the GAR domain of SNAP1 is 5X57, which is the structure of the GAR domain of the human protein ACF7 (43). The predicted structure of the entire protein for SNAP1, NHL1, and TbSpef1 was obtained from the AlphaFold protein structure database (https://alphafold.ebi.ac.uk/).

### Data analysis and statistical analysis

The ImageJ software (National Institutes of Health, Bethesda, MD; http://imagej.nih.gov/ij/) was used to measure the distance between flagellar pocket collars and between basal bodies, the length of the FAZ, and the fluorescence intensity of NHL1-3HA and SNAP1-3HA in control and RNAi cells. Data were exported to GraphPad Prism 9 for analysis. Statistical analysis was conducted using the two-tailed Student’s *t* test or Chi-square test. Error bars represented standard deviation (SD) from three biological replicates.

## Data availability

All data are contained within the manuscript.

## Supporting information

This article contains supporting information.

## Conflicts of interest

The authors declare that they have no conflicts of interest with the contents of this article.

## Supporting information

Supporting Figure S1 **Proximity ligation assays to detect the *in situ* protein-protein interaction among SNAP1, NHL1, and TbSpef1.***A*, PLA experiments using 29-13 (no tagged proteins expressed), 29-13 cells expressing PTP-tagged NHL1, 29-13 cells expressing 3HA-tagged SNAP1, and 29-13 cells co-expressing 3HA-tagged SNAP1 and PTP-tagged NHL1 or co-expressing PTP-tagged SNAP1 and 3HA-tagged TbSpef1. Please note that the images labeled as “SNAP1-3HA + PTP-NHL1” and “SNAP1-PTP + TbSpef1-3HA” in panel (*A*) are the same images presented in Figures 6*A* and 7A, because this supplemental figure is the complete data set of the PLA assays performed for Figures 6*A* and 7A. Scale bar: 5 μm. *B*, Quantitation of the cells with a positive PLA signal at the proximal end of the MtQ in the cells presented in panel (*A*). Error bars indicate S.D. from three biological replicates. ∗∗∗∗*p* <0.0001 (Chi-square test).
