# Supplementary figures and images for: The microtubule quartet protein SNAP1 in Trypanosoma brucei facilitates flagellum and cell division plane positioning by promoting basal body segregation
Source: J Biol Chem. 2023 Oct 12;299(11):105340. doi: 10.1016/j.jbc.2023.105340 (PMC10656233; doi:10.1016/j.jbc.2023.105340)

Figure S1

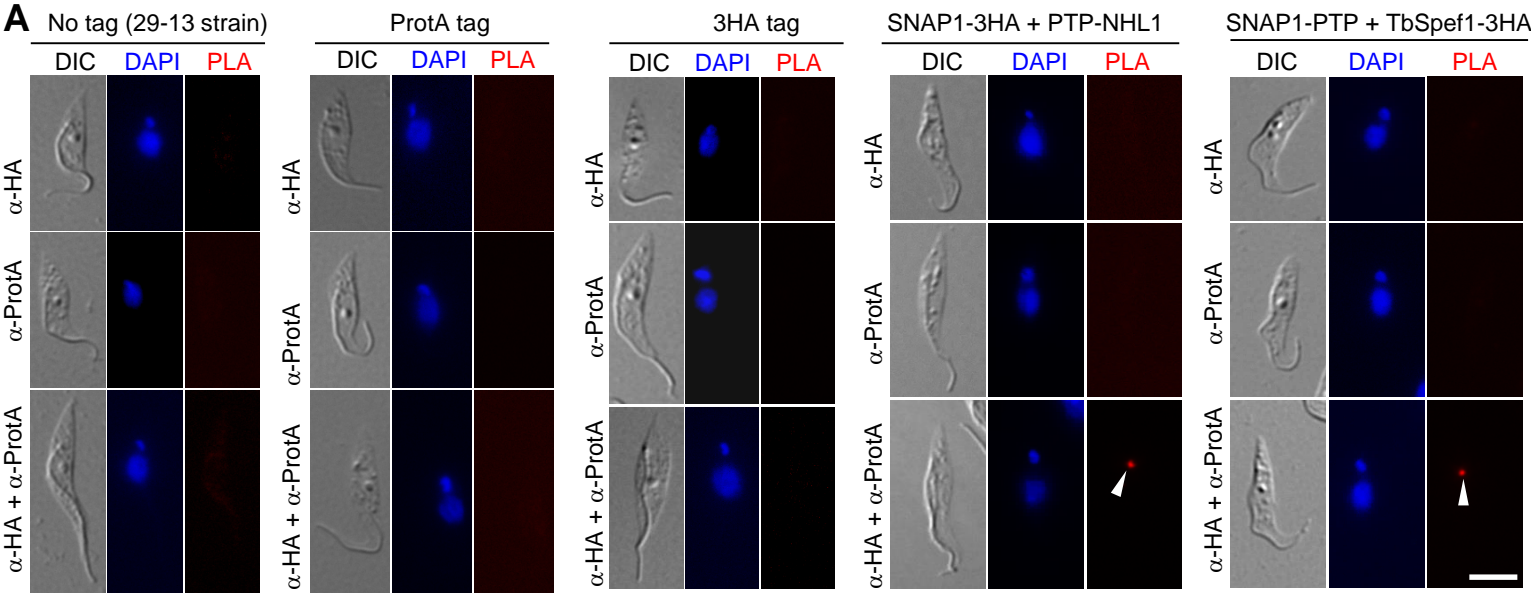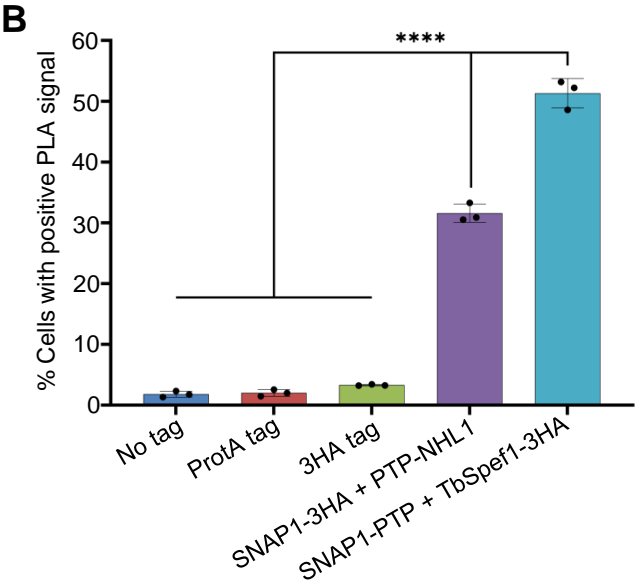

Supplement: Supporting Figure S1 — Proximity ligation assays to detect the in situ protein-protein interaction among SNAP1, NHL1, and TbSpef1.A, PLA experiments using 29-13 (no tagged proteins expressed), 29-13 cells expressing PTP-tagged NHL1, 29-13 cells expressing 3HA-tagged SNAP1, and 29-13 cells co-expressing 3HA-tagged SNAP1 and PTP-tagged NHL1 or co-expressing PTP-tagged SNAP1 and 3HA-tagged TbSpef1. Please note that the images labeled as “SNAP1-3HA + PTP-NHL1” and “SNAP1-PTP + TbSpef1-3HA” in panel (A) are the same images presented in Figures 6A and 7A, because this supplemental figure is the complete data set of the PLA assays performed for Figures 6A and 7A. Scale bar: 5 μm. B, Quantitation of the cells with a positive PLA signal at the proximal end of the MtQ in the cells presented in panel (A). Error bars indicate S.D. from three biological replicates. ∗∗∗∗p <0.0001 (Chi-square test). [file mmc1.pdf]
